# Effect of additive cellulase on fermentation quality of whole-plant corn silage ensiling by a *Bacillus* inoculant and dynamic microbial community analysis

**DOI:** 10.3389/fmicb.2023.1330538

**Published:** 2024-01-09

**Authors:** Xudong Liu, Aifang Wang, Liqi Zhu, Wei Guo, Xiaojun Guo, Baocheng Zhu, Ming Yang

**Affiliations:** ^1^College of Life Sciences, Hebei Agricultural University, Baoding, China; ^2^College of Horticulture, Hebei Agricultural University, Baoding, China; ^3^Hebei Engineering Research Center for Agricultural Waste Resource Utilization, Baoding, China

**Keywords:** whole-plant corn silage, *Bacillus* inoculant, cellulase, fermentation, microbial community

## Abstract

Whole-plant corn silage (WPCS) has been widely used as the main roughage for ruminant, which promoted the utilization of corn stover for animal feed production. However, rigid cell wall structure of corn stover limits the fiber digestion and nutrients adsorption of WPCS. This study investigated the effect of adding cellulase on improving the fermentation quality of WPCS ensiling with a *Bacillus* complex inoculant. With the *Bacillus* (BA), the lactic acid accumulation in the WPCS was significantly higher than that in control (CK). The additive cellulase (BC) increased the lactic acid content to the highest of 8.2% DW at 60 days, which was significantly higher than that in the CK and BA groups, and it reduced the neutral detergent fiber (NDF) and acid detergent fiber (ADF) contents from 42.5 to 31.7% DW and 28.4 to 20.3% DW, respectively, which were significantly lower than that in the CK and BA groups. The crude protein and starch were not obviously lost. Dynamic microbial community analysis showed that the *Bacillus* inoculant promoted the lactic acid bacteria (LAB) fermentation, because higher abundance of *Lactobacillus* as the dominant bacteria was observed in BA group. Although the addition of cellulase slowed the *Lactobacillus* fermentation, it increased the bacterial community, where potential lignocellulolytic microorganisms and more functional enzymes were observed, thus leading to the significant degradation of NDF and ADF. The results revealed the mechanism behind the degradation of NDF and ADF in corn stover, and also suggested the potential of cellulase for improving the nutritional quality of WPCS.

## 1 Introduction

In recent years, the development of whole-plant corn silage (WPCS) on a large scale has greatly promoted the utilization of corn stover for animal feed production (Ferraretto et al., 2018; Cui et al., 2022). To produce high quality WPCS, silage inoculants are usually applied to enhance the fermentation process, thus leading to a change in palatability, inhibition of aerobic spoilage, and nutrient preservation (Muck et al., 2018). The silage inoculants have been divided into 6 categories: homofermentative lactic acid bacteria (LAB), obligate heterofermentative LAB, combination inoculants containing obligate heterofermentative LAB plus homofermentative LAB, and other inoculants, chemicals, and enzymes (Muck et al., 2018).

LAB are the most commonly used additives during fermentation to improve the ensiling process of silages (Muck et al., 2018). However, LAB are probably insufficient to improve the nutritional quality of silages and animal performance. The corn stover in WPCS has a rigid cell wall structure formed by cellulose, hemicellulose and lignin, thus limiting the fiber digestion and nutrients adsorption in ruminants *in vivo* (Guo et al., 2022). As reported earlier, the digestibility of WPCS in the rumen of ruminants is usually less than 60% and is even lower when the WPCS is harvested late (Raffrenato et al., 2017). To improve the nutritional value of WPCS, microbes or enzymes that can destroy the cell wall of corn stover are necessary.

*Bacillus*, as a kind of silage inoculant, has the potential to improve the nutritional quality of silages since it can produce a broad spectrum of lignocellulolytic enzymes, thus leading to the degradation of lignocellulose, and it also has the characteristics of strong resistance to adverse environments (Guo et al., 2022). *Bacillus* has also been proven to improve the fermentation quality of silages and inhibit their aerobic spoilage (Lara et al., 2015; Bai et al., 2020). In addition, as a biological control agent, it could effectively protect plants against plant pathogens (Arrebola et al., 2010; Zhao et al., 2014). It could also be used as a feed supplement directly or as a bacterial inoculant in biological feeds for monogastric animals, such as poultry and pigs (Shi et al., 2017; Zhang et al., 2018; Li et al., 2020).

Compared with microbial fermentation, exogenous cellulase can also degrade structural carbohydrates of the cell wall to soluble sugars, which are substrates for LAB fermentation (Wang et al., 2002; Colombatto et al., 2004). Thus, the interaction of cellulase with LAB to improve the quality of silages has been extensively studied, and the advantages in increasing dry matter, crude protein and lactic acid contents, and decreasing acid detergent fiber (ADF) and neutral detergent fiber (NDF) contents have been verified. The silages include fresh rice straw silage (Sarwono et al., 2021), oat silage (Xu et al., 2022), *Caragana korshinskii* silage (Bai et al., 2023), mulberry leaf silage (*Lactobacillus*) (He et al., 2019), a mixture of amaranth and rice straw (*Lactobacillus plantarum*) (Mu et al., 2020), and mixed silage of whole-plant corn and peanut Vines (Wang et al., 2022).

As far as we understand, cellulase is not sufficient for the effective degradation of lignocellulose, and the synergy of cellulase with other enzymes, such as xylanase and feruloyl esterase (Wang et al., 2022), or with the corresponding enzyme-producing bacteria could be more effective (Zhang et al., 2022). To date, few studies have focused on improving the fermentation quality of WPCS by cooperation of *Bacillus* and cellulase inoculants. We hypothesize that they are effective for the degradation of lignocellulose, decreasing the contents of neutral detergent fiber (NDF) and acid detergent fiber (ADF), thus leading to the improvement of the nutritional quality of WPCS. In this study, we investigated the effect of adding cellulase with a *Bacillus* complex inoculant on the degradation of NDF and ADF, the accumulation of organic acids, and the nutrient contents in WPCS. Furthermore, the bacterial community was also analyzed to provide insight into the improvement of fermentation by the *Bacillus* complex inoculant and cellulase.

## 2 Materials and methods

### 2.1 Silage inoculants

*Bacillus* complex inoculant, composed of *B. amyloliquefaciens* and *B. subtilis*, was provided by Zhongbang Biotechnology Co. LTD (Baoding, China). The commercial cellulase Cellic^®^ CTec2 (Novozymes A/S, Bagsværd, Denmark) was used as the cellulase preparation.

### 2.2 Silage production

Whole-plant corn was harvested on the 10th of September 2021 in the area surrounding Baoding, China. The harvested whole-corn plant was sliced off and transported to the feed processing site of the Hebei Province Feed Microorganism Technology Innovation Center and then chopped to 1–2 cm in size with a forage cutter (Baoding Golden Land Ecological Engineering Co., Ltd., Baoding, China).

*Bacillus* silage inoculant was added at a dosage of 9 log10 CFU/kg (fresh weight) to chopped whole-plant corn, which was used as the treatment group (BA). The same amount of distilled water was added to the whole-plant corn and designated as the control group (CK). The cellulase was added with the dosage of 500 FPU/kg on the basis of the ensiling by the *Bacillus* silage inoculant (BC group).

Whole-plant corn of the CK, BA, and BC groups was put into 30 cm × 40 cm polyethylene plastic bags in 1.0 kg amounts, and the bags were vacuum-sealed. There were 20 silos for each treatment, stored at room temperature (25 ± 2°C), and 3 silos were opened and sampled after 2, 15, 30, and 60 days of ensiling. The WPCS taken from each treatment was divided into two portions. One portion was used to evaluate the quality of the silage by measuring the fermentation parameters including the pH, organic acids, nutrients, and the NDF and ADF contents. The other portion was stored in a −80°C refrigerator immediately and used for microbial diversity analysis.

### 2.3 pH and organic acid analyses

For the pH and organic acid analyses, a total of 20 g samples were homogenized in 180 mL of distilled water, stirred for 30 min and then filtered through 4 layers of medical gauze. The pH value of the filtrate was measured using a pH meter (E-201-D, Shanghai Yidian Scientific Instrument Co., Ltd., China). The lactic acid, acetic acid, and butyric acid in the filtrate were analyzed using a high-performance liquid chromatography machine (Agilent 1260, Agilent Technologies Inc., United States). Experimental conditions: SB-AQ C18 column (4.6 mm × 250 mm); mobile phase A (methanol): mobile phase B (0.01 mol/L (NH4)_2_HPO_4_, pH = 2.70) = 3:97; flow rate was 1.0 mL/min; injection volume was 20 μL; detection wavelength was 210 nm; and column temperature was 25°C.

### 2.4 Nutrient analyses

The dry matter (DM) contents of the WPCS were calculated based on the fresh weight and dry weight after oven-drying at 65°C for 72 h. After drying, the samples were ground through a 1 mm screen using a waring blender for nutrient analyses. The nitrogen content was determined using a Kjeldahl apparatus (K9860, Automated Analyzer, Hanon Instruments, China), and the crude protein (CP) was calculated by multiplying the nitrogen content by 6.25. The NDF and ADF were determined according to the methods using a full-automatic fiber meter (A2000i, ANKOM, United States) (Van Soest et al., 1991). The starch was determined by means of polarimetry (Polax 2 L, Atago^®^, Tokyo, Japan).

The lignin content was measured according to the NREL method (Sluiter et al., 2012). Materials weighing 300 mg were treated with 72% H_2_SO_4_ for 1 h at 30°C in a 100-mL triangular flask and then diluted to 4% H_2_SO_4_ with deionized water and autoclaved for 1 h at 121°C. The slurry was vacuum filtered through filtering crucibles. The filtrate and residue were used for acid soluble lignin and acid insoluble lignin determination, respectively. The total lignin content was calculated. The monosaccharide (ESC) content was analyzed with the 3,5-dinitrosalicylic acid method (Miller, 1959).

### 2.5 Bacterial community analyses

Silage samples weighing 10.0 g were put into sterilized eluent (0.9%NaCl +0.1%Tween-80) and blended at 100 rpm for 2 h, and then 4 layers of gauze were used to filter the supernatant. The precipitate was obtained by centrifugation at 5000 rpm for 15 min. The total genomic DNA of the microbes was extracted from the precipitate by using a DNA extraction kit (TIANGEN, Beijing). The quality and concentration of the DNA were detected using a Nanodrop 2000 spectrophotometer (Thermo Scientific, Wilmington, SC, United States).

The bacterial 16S rRNA genes were amplified via PCR using the primers 340F (5′-CCTACGGGNBGCASCAG-3′) and 805R (5′-GACTACNVGGGTAT CTAATCC-3′). The bacterial 16S amplicon was sequenced with a Hiseq2500 (Illumina, San Diego, CA, United States) according to a previous description (Guo et al., 2019). Quality-filter and cluster analyses were performed. The 16S rRNA sequencing data were analyzed using the online Majorbio Cloud Platform (Shanghai Majorbio Bio-Pharm Technology Co., Ltd., China).

All sequences were homologously clustered into operational taxonomic units (OTUs) based on 97% sequence similarity by using the QIIME UCluster method. To determine the species classification information corresponding to each OTU, the RDP classifier Bayesian algorithm was used for the taxonomic analysis of representative OTU sequences with 97% similarity, and they were then compared to the 16S rRNA database. The community species composition of each sample was measured at the Domain, Kingdom, Phylum, Class, Order, Family, Genus, and Species levels. The microbial diversity in the silage was analyzed according to the α diversity index, including the Shannon index, Chao1 index, Simpson index, Ace index and community coverage index. The differences in bacterial proportion between the groups at genus level were analyzed using one-way ANOVA. Bacterial function prediction was verified from the Kyoto Encyclopedia of Genes and Genomes (KEGG) database using Phylogenetic Investigation of Communities by Reconstruction of Unobserved States (PICRUSt2), which predicts the functional abundance of samples based on the abundance of marker gene sequences in the sample.

### 2.6 Statistical analyses

The effects of the treatments on fermentation parameters, chemical composition were analyzed by means of a mixed linear model (procedure MIXED in SPSS 15.0.1, SPSS Inc., Chicago, IL). The model used was y = μ + treatment+time + treatment×time + ε, where μ is a constant. The “treatment” (i.e., BA and BC) and “time” (i.e., ensiling days) were regarded as fixed factors and ε as a random term. The significance of the difference between the treatments at different ensiling days was tested by contrasts using Bonferroni-corrected significance levels. The number of replicate samples was three.

## 3 Results and discussion

### 3.1 Changes in pH and organic acid contents during ensiling

**Figure 1:**
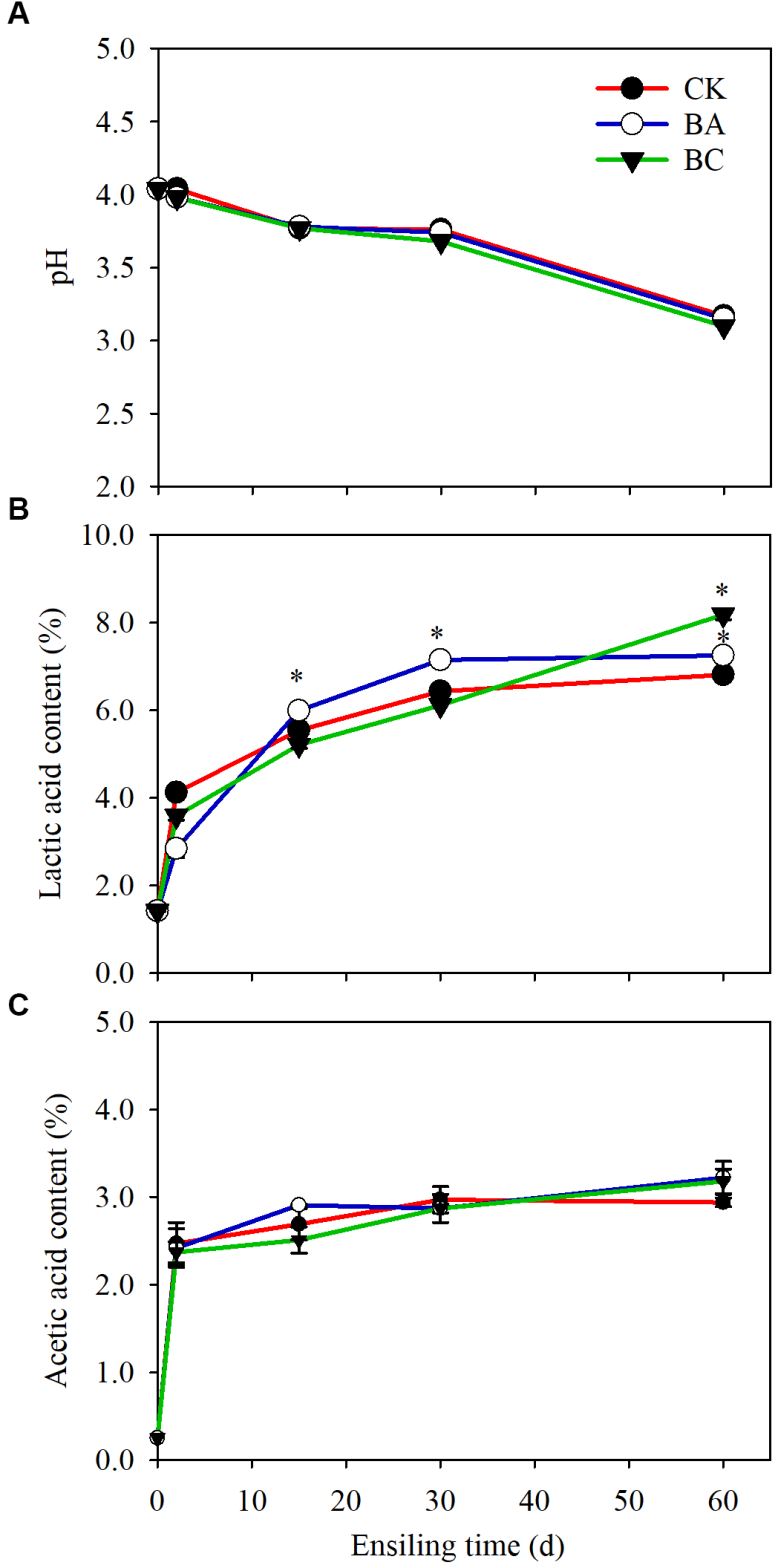
The pH **(A)**, lactic acid content **(B)** and acetic acid content **(C)** in WPCS in CK, BA and BC groups during the ensiling process. The asterisks indicate the significant differences level of the samples compared to CK, *p ≤ 0.05.

The pH values of WPCS in both the control (CK) and treatment groups (BA and BC) started to decrease after 2 days of ensiling and then decreased rapidly after 30 days. At 60 days, the pH of the samples in the BC group decreased to 3.1, which was slightly lower than that in the CK and BA groups (Figure 1A). The decrease in pH values was due to the accumulation of organic acids, including lactic acid and acetic acid (Figures 1B,C). Butyric acid was not observed in the three groups. The percentages of lactic acid increased with the extension of ensiling time (Figure 1B). In CK, 6.4% lactic acid in dry weight (DW) was observed after 30 days, and then it increased slightly to 6.8% until 60 days. The content of lactic acid in BA was significantly higher (*p* ≤ 0.05) starting from 15 days than that in CK, and 7.1% was accumulated after 30 days. In the BC groups, the lactic acid content was lower than that in the CK and BA groups within 30 days, but it was significantly higher (*p* ≤ 0.05), reaching 8.2% after 60 days. There were no significant differences in the accumulation of acetic acid between CK and the treatment groups within 30 days, but at 60 days, the contents of acetic acid in BA and BC were slightly higher than that in CK (Figure 1C).

The results suggested that considerable organic acids accumulated in the CK group without using the silage inoculants. As reported earlier, anaerobic environment is conducive to the fermentation of LAB that are attached to the plant surface (Bai et al., 2020). The lactic acid content in the BA group was higher than that in the CK group, indicating that the *Bacillus* inoculant probably helped to consume the remaining oxygen during the early stages of ensiling, thus creating a better anaerobic environment for *Lactobacillus* fermentation (Bai et al., 2020). Interestingly, the lactic acid content in the BC group exceeded that in the CK and BA groups after 60 days, which could be due to the effect of additive cellulase. The cellulase possibly increased the degradation of lignocellulose to the fermentable sugars for the growth of multiple bacteria, which slightly suppressed the LAB growth within 30 days, and with the time extension to 60 days, the LAB still maintained fermentation. Thus, the investigation of the microbial community could possibly explain the dynamic changes in lactic acid contents. Acetic acid can be produced by heterofermentative LAB, that improving the aerobic stability of silages (Xu et al., 2019), but also can be formed by the degradation of acetyl groups from hemicellulose (Jönsson and Martín, 2016), because the pH values in these three groups were all low, and acidic conditions led to the degradation of hemicellulose and further to acetic acid.

### 3.2 Dynamic changes in chemical compositions

**Figure 2:**
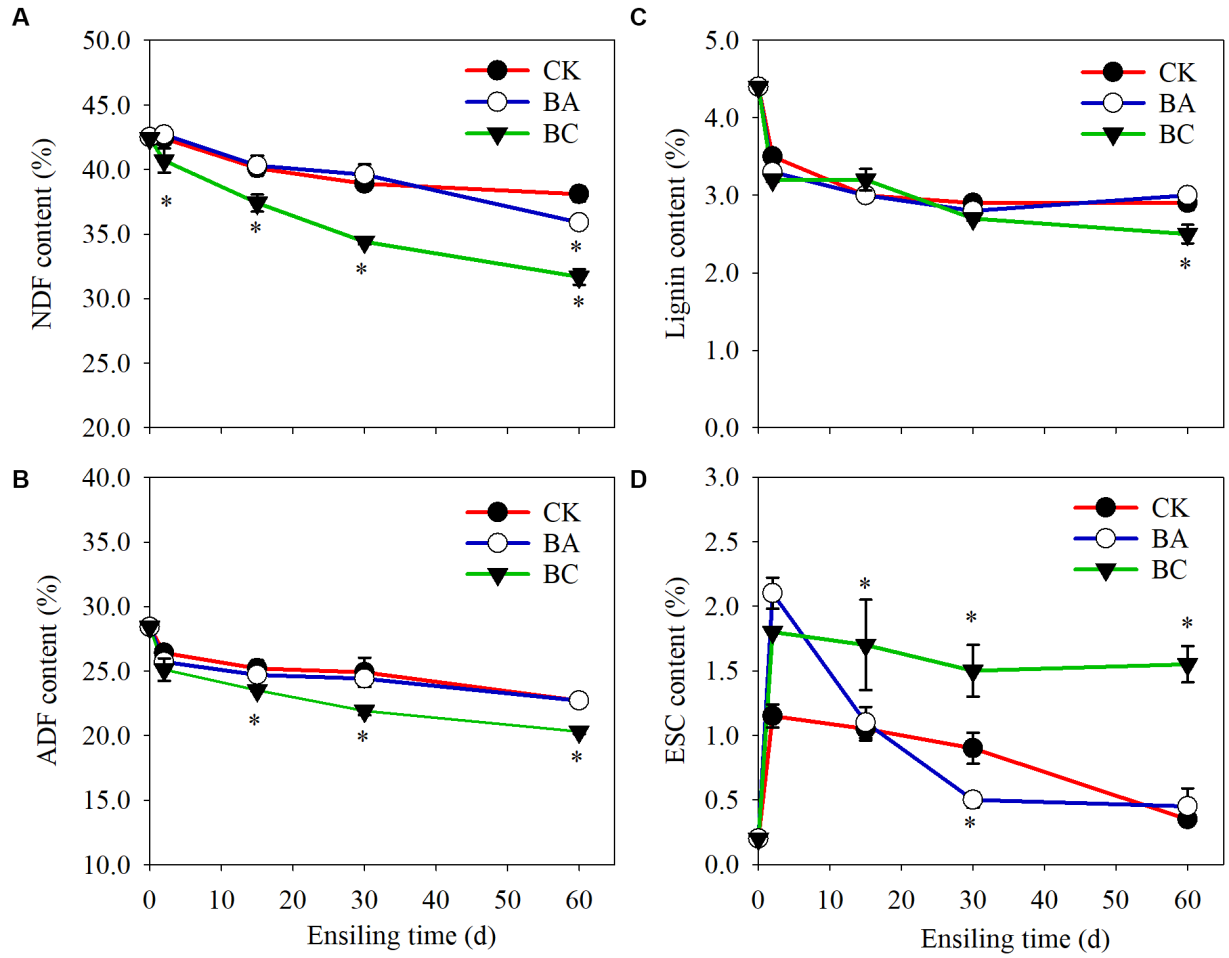
The contents of neutral detergent fiber (NDF) **(A)**, acid detergent fiber (ADF) **(B)**, lignin **(C)** and monosaccharide (ESC) **(D)** in WPCS in CK, BA, and BC groups during the ensiling process. The asterisks indicate the significant differences level of the samples compared to CK, *p ≤ 0.05.

NDF and ADF are important indicators to evaluate the quality of roughage. It is generally acknowledged that the NDF determines DM intake, and the higher the NDF content is, the lower the intake. The ADF affects the digestibility and energy of roughage. Thus, the lower the ADF is, the better. The NDF and ADF contents in both the control and treatment groups decreased during the whole ensiling process (Figure 2). In CK group, the NDF content decreased to 38.1% DW after 60 days of ensiling. In BA group, the NDF content decreased to 35.9%. However, it is worth noting that the NDF content in the BC group decreased to 31.7%, which was significantly lower (*p* ≤ 0.05) than that in both the CK and BA groups (Figure 2A). There were no significantly differences in ADF contents in CK and BA groups (Figure 2B). However, in BC group, the ADF contents were significantly lower (*p* ≤ 0.05) than those in the CK and BA groups after 2 days of ensiling.

The results indicated that the lignocellulose in corn stover degraded during the ensiling process, and the addition of the *Bacillus* inoculant exhibited the positive effect during the late fermentation period after 30 days, particularly on NDF removal. Significant lignocellulose degradation was observed in the BC group than that that in the BA group, indicating improvements in the fermentation quality of WPCS by the addition of cellulase. The decrease in NDF and ADF contents could be contributed to two processes, including chemical and enzymatic hydrolysis. As described earlier, the accumulated organic acids can degrade lignocellulose (Larsen et al., 2017). On the other hand, cellulase or microbes (*Bacillus* and other microbes) produce cellulase, hemicellulase or other enzymes such as feruloyl esterase can hydrolyze the structural carbohydrates in plants during silage fermentation, which has been widely reported (Ning et al., 2017). The further decrease in NDF content in the BA group was probably because of the function of *Bacillus*. In the present study, the *Bacillus* inoculant contained *B. subtilis* and *B. amyloliquefaciens*, which have been reported to be capable of degrading lignocellulose (Li et al., 2014, 2015; Tian et al., 2017). This is also in agreement with the study of both WPCS and alfalfa silage ensiling by *Bacillus* inoculation (Bai et al., 2020, 2022).

Additive cellulase promoted the greatest degradation degree of WPCS, which is in agreement with studies using cellulase as an additive inoculant (Mu et al., 2020; Bai et al., 2023). Additionally, we found that the lignin content in the CK, BA and BC groups decreased after 2 days of ensiling, and remained stable in the following process, and the lignin content in the BC group was significantly lower than that in CK and BA groups after 60 days (Figure 2C), indicating that the degradation of lignocellulose was not only contributed by cellulase but also by microbes or their synergistic effect. The monosaccharide (ESC) content in CK was maintained at a low level and that in the BA group increased after 2 days but decreased rapidly in the following days (Figure 2D). In the BC group, the ESC increased after 2 days and was maintained in the following days. It is acknowledged that ESC can be carbon source for the growth of microbes. The relatively high level of ESC in the BC group probably promoted the growth of *Bacillus* or other microbes.

The contents of dry matter (DM), starch and crude protein in the raw WPCS were 41.0, 33.4, and 9.2% DW, respectively. After 2 days of ensiling, the DM contents in the three groups started to decrease (Table 1), and the DM contents in BC groups were significantly lower (*p* ≤ 0.05) than that in CK group, which confirmed the considerable degradation of NDF and ADF in BC group. The starch contents in the three groups decreased at the early stage of ensiling (2 or 15 days), and then increased during the late stage of the ensiling (30 or 60 days), which was probably due to the relative decrease of NDF and ADF contents. There were no obvious changes in crude protein content. Generally, there were no considerable differences in the contents of such nutrients between the three groups, indicating that the addition of *Bacillus* and cellulase inoculant did not lead to the loss of nutrient components during the ensiling process.

**Table 1:** Changes in the contents of dry matter (DM), starch and crude protein (CP) in CK, BA, and BC groups during the ensiling process.

Items (%)	Groups	Ensiling days				*p* value		
		2	15	30	60	T	D	T × D
DM	CK	38.9 ± 0.06	38.5 ± 0.09	37.8 ± 0.09	36.2 ± 0.03	0.179	0.000	0.003
	BA	39.9 ± 0.33	38.9 ± 0.22	37.4 ± 0.03	36.1 ± 0.06			
	BC	39.7 ± 0.48	38.1 ± 0.40	36.5 ± 0.28*	35.2 ± 0.14*			
Starch	CK	31.2 ± 0.34	31.6 ± 0.70	34.4 ± 1.18	30.5 ± 0.03	0.730	0.000	0.000
	BA	33.6 ± 0.20*	30.2 ± 0.23	30.6 ± 0.15*	32.8 ± 0.20*			
	BC	30.5 ± 0.44	30.6 ± 0.38	33.7 ± 0.14	34.0 ± 0.04*			
CP	CK	9.4 ± 0.09	9.1 ± 0.17	9.2 ± 0.09	9.4 ± 0.03	0.443	0.000	0.000
	BA	10.3 ± 0.03*	9.4 ± 0.17	9.0 ± 0.06	9.0 ± 0.00			
	BC	9.5 ± 0.14	9.7 ± 0.12	9.0 ± 0.06	9.3 ± 0.14			

T, treatment; D, ensiling days; T × D, the interaction between the treatment and ensiling days. The asterisks indicate the significant differences level of the samples compared to CK, *p ≤ 0.05.

### 3.3 Dynamic changes in bacterial communities during ensiling

#### 3.3.1 Alpha diversity

To gain insight into the WPCS ensiling process, the dynamic changes in bacterial communities in the CK, BA and BC groups were analyzed. In total, 2,041,555 quality-filtered sequences of 16S rRNA were acquired from 36 samples and clustered into 1,051 OTUs. The Good’s coverage was approximately 0.99 in all treatments (Table 2).

**Table 2:** Richness and diversity indices of microbial communities in CK, BA, and BC groups during the ensiling process.

Samples	Sequence number	OTUs	Shannon	Simpson	Ace	Chao 1	Coverage
CK2d	51,076	266	2.550	0.146	414.26	402.14	0.9976
BA2d	46,142	218	2.325	0.155	328.63	284.85	0.9971
BC2d	48,198	231	2.450	0.151	319.24	302.05	0.9973
CK15d	40,027	264	2.322	0.191	367.19	384.32	0.9980
BA15d	43,172	233	2.098	0.223	357.51	332.73	0.9966
BC15d	43,112	255	2.467	0.158	340.46	351.32	0.9984
CK30d	37,088	315	2.265	0.290	452.83	414.33	0.9980
BA30d	37,377	263	2.114	0.303	408.77	381.81	0.9969
BC30d	40,389	484	2.755	0.224	611.50	614.61	0.9979
CK60d	43,013	338	2.942	0.125	437.37	420.18	0.9980
BA60d	42,693	297	2.701	0.155	374.50	369.02	0.9975
BC60d	37,066	286	2.656	0.206	336.12	337.83	0.9980

**Figure 3:**
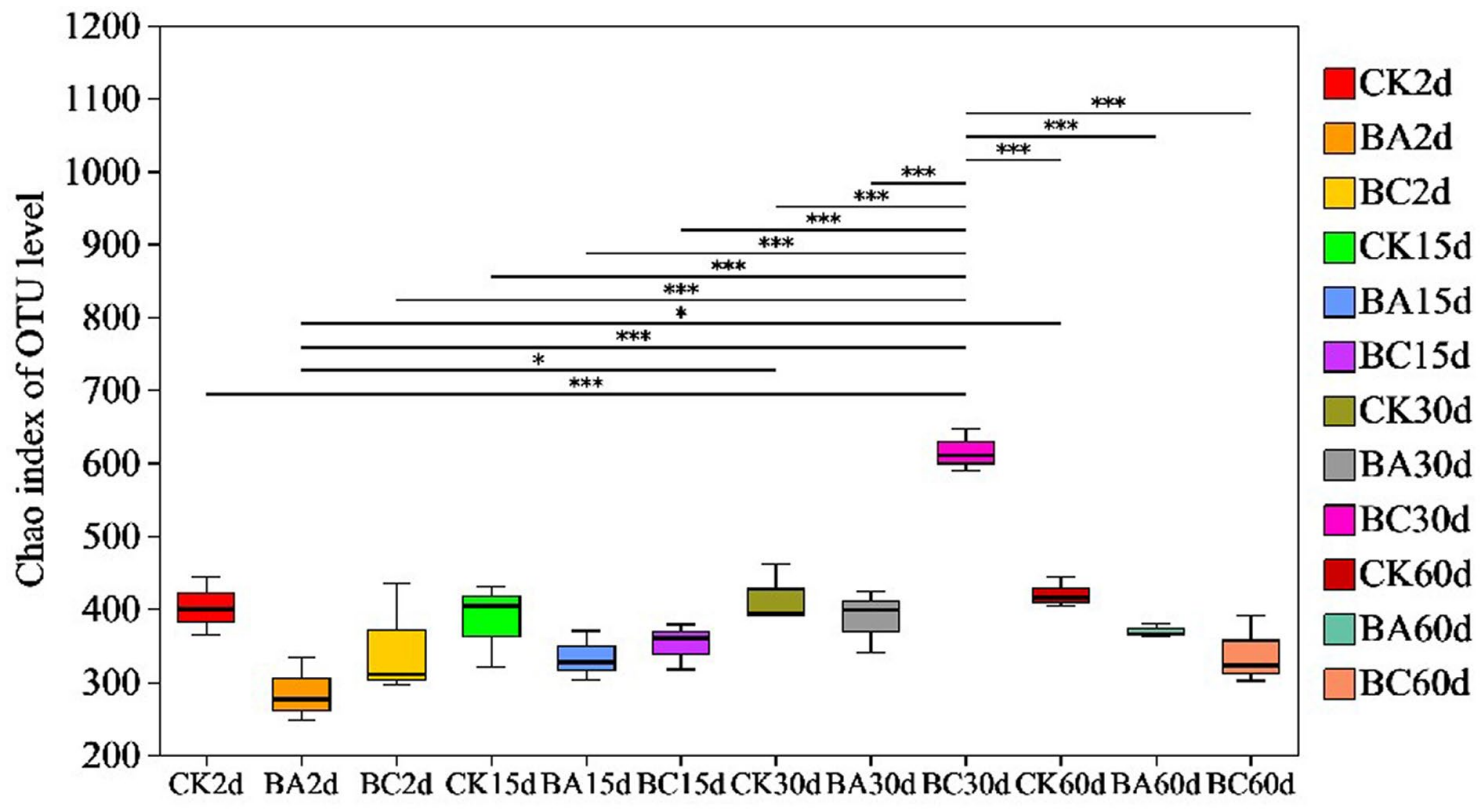
Differences in bacterial community diversity and richness in Chao index in CK, BA, and BC groups during the ensiling process. The asterisks indicate the significant differences level between the samples, *p ≤ 0.05, **p ≤ 0.01, ***p ≤ 0.001.

According to Chao1 index, the alpha diversity of bacteria during the ensiling process showed no obvious differences in the abundance of the bacterial community in the WPCS after 2 and 15 days of ensiling (Figure 3). However, after 30 days, the abundance of WPCS in the BC group significantly increased, and they were returned back to a consistent level after 60 days. The similar tendency was observed in Ace and Shannon indexes, and the Simpson index showed the contrary tendency (Table 2). It has been reported that when LAB became the dominant bacteria, the alpha diversity decreased (Xu et al., 2020). It was true that the alpha diversity decreased compared to the samples without ensiling (Xu et al., 2020). However, according to our results, it seems that significantly higher alpha diversity in the BC group after 30 days of ensiling was observed. The reason could be explained by the relatively higher amount of ESC for the growth of different bacteria (Figure 2D). Thus, it is interesting to see the bacterial community of the WPCS to explain the effect of *Bacillus* inoculant and the additive cellulase.

#### 3.3.2 Bacterial community

**Figure 4:**
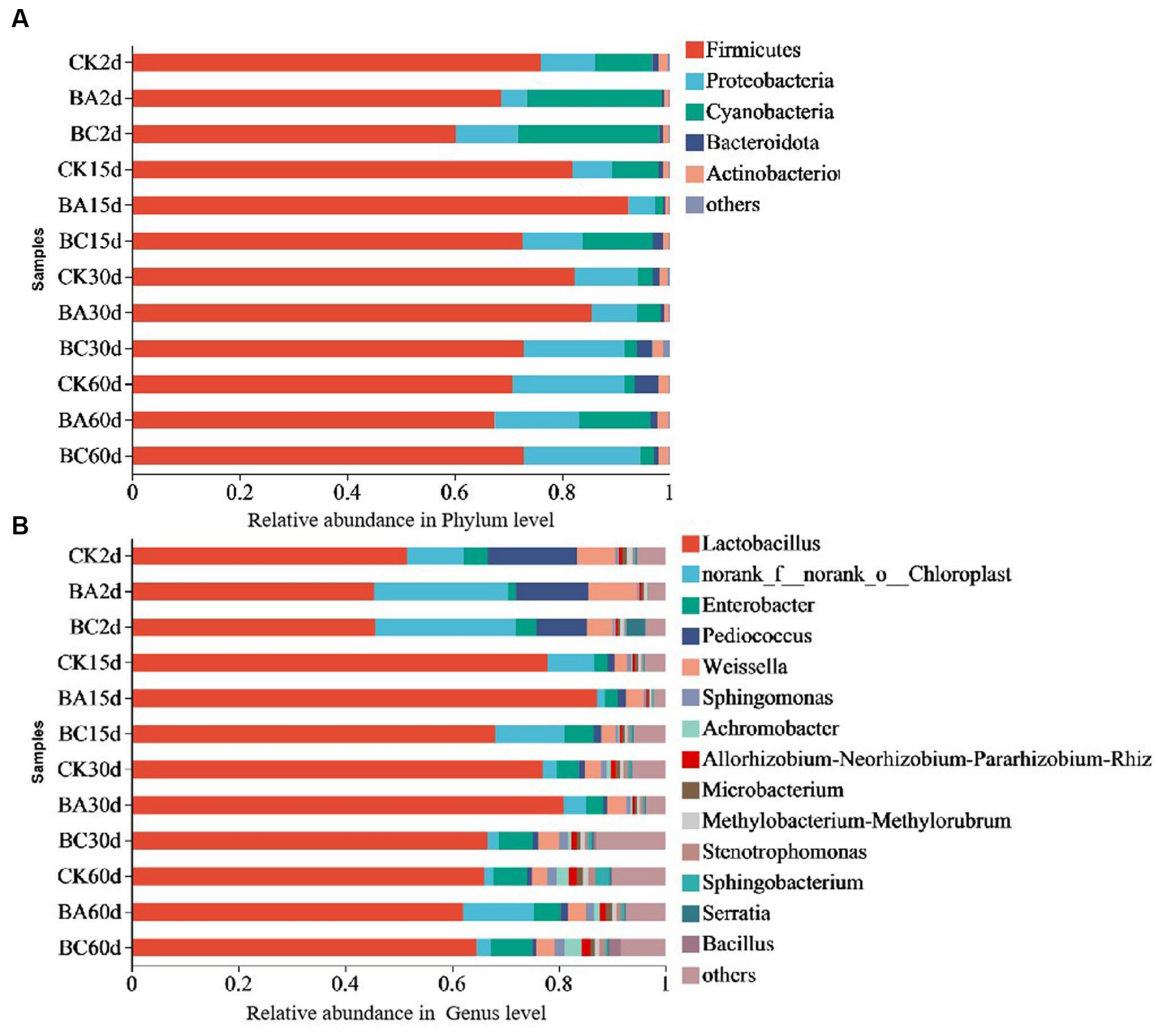
Relative bacteria abundance at phylum **(A)** and genus **(B)** levels in CK, BA and BC groups during the ensiling process.

The bacterial community dynamics during the ensiling process of the WPCS were observed (Figure 4). At the phylum level, the dominant phyla of the CK, BA and BC groups were Firmicutes, Proteobacteria, and Cyanobacteria (Figure 4A). Among them, Firmicutes was the most predominant phylum. Basically, the abundance in the CK group was higher than that in the BA and BC groups after 2 days. However, after 15 days until 30 days, it was higher in the BA group than that in the CK and BC groups. After 60 days, they were at similar levels. During the whole ensiling process, the abundance of Proteobacteria gradually increased, and the abundance of Cyanobacteria decreased in the three groups. Additionally, Bacteroidota and Actionobacteriota were also annotated, and their abundances in BC30d and CK60d were relatively higher than those in the other groups.

The compositions of bacterial communities in the WPCS at the genus level were identified (Figure 4B). The dominant genus in all samples was *Lactobacillus*, followed by *Enterobacter*, *Pediococcus*, and *Weissella*. Similar to Firmicutes, the abundance of *Lactobacillus* increased after 15 days of ensiling, and it was higher in the BA group until 30 days. After 60 days, the levels were similar in the three groups. It is difficult to find a clear pattern of change in the abundances of *Enterobacte*, *Pediococcus*, and *Weissella* in the three groups during the whole ensiling process. The relative abundance of *Bacillus* was very low in the samples at 2 and 15 days. However, in the BC group, it started to increase after 30 days and to a certain level at 60 days.

The results indicated that Firmicutes became the most predominant phylum as the silage process progressed, and the most effective genus was *Lactobacillus*, which is consistent with the studies of alfalfa silage fermentation (Yang et al., 2019; Bai et al., 2020). This verified the reason for the increase in the lactic acid content and decrease in the pH values (Guan et al., 2018; Yang et al., 2019) The *Lactobacillus* abundance increased in the BA group after 15 and 30 days of ensiling, indicating the positive effect of the *Bacillus* inoculant on creating a better anaerobic environment for *Lactobacillus* fermentation. This could also explain the higher lactic acid content in BA group than the other groups (Figure 1A). The addition of cellulase did not increase *Lactobacillus* abundance; however, it increased the growth of *Bacillus* and other bacteria after 30 days of ensiling, which would be helpful for the degradation of lignocellulose. Considering the effect of *Bacillus* on lignocellulose degradation, the increased abundance of *Bacillus* probably played a role in improving silage quality from the point of view of lignocellulose degradation (Guo et al., 2022).

#### 3.3.3 Bacterial proportion

**Figure 5:**
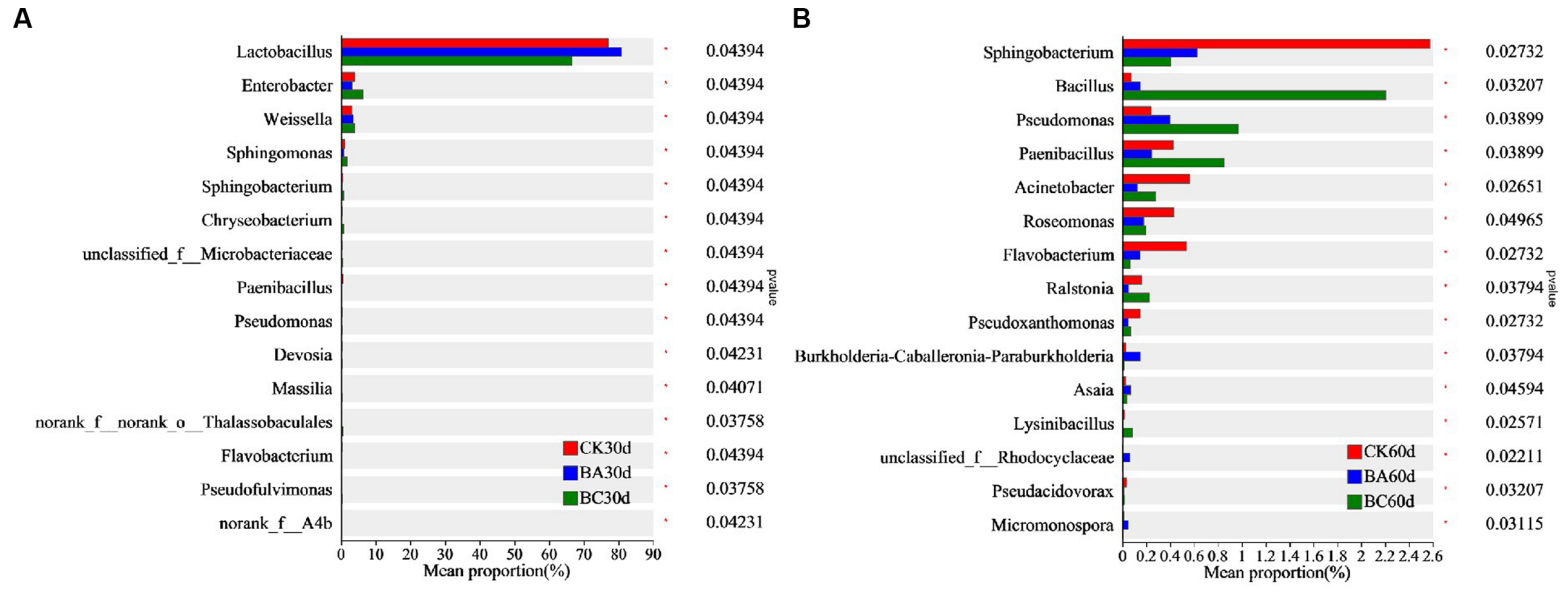
Comparison of different bacteria among the CK, BA, and BC groups after 30 days **(A)** and 60 days **(B)** ensiling. The asterisks indicate the significant differences level of between the samples, *p ≤ 0.05.

The differences in bacterial proportion among the three groups at genus level were analyzed using one-way ANOVA (Figure 5). After 30 days of ensiling, the proportion of *Lactobacillus* in the BA group was significantly higher than that in the CK group, and it was much lower in the BC group (Figure 5A). However, the proportions of *Enterbacter*, *Weissella*, *Sphingomonas*, *Sphingobacterium*, and *Chryseobacterium* in the BC group were higher than those in the other two groups. In addition, bacteria, including *Pseudomonas*, *Massilia*, and *Pseudofulvimonas*, were also significantly higher in the BC group. After 60 days of ensiling, the abundance of *Bacillus*, *Pseudomonas*, and *Paenibacillus* in the BC group were significantly higher than those in the other two groups (Figure 5B). The proportions of *Sphingobacterium*, *Acinetobacter*, *Roseomonas*, and *Flavobacterium* in the CK group were relatively higher. It is worth noting that the proportion of *Bacillus* in the BA group was significantly higher than that in CK but lower than in that in the BC group.

The results showed that in addition to LAB, more other bacterial genera were observed in the BC group after 30 and 60 days of ensiling, indicating the promotion effect of additive cellulase on bacterial growth. In these bacterial genera, some bacteria have been reported to be capable of degrading lignocellulose. *Enterobacter* sp. SUK-Bio produced cellulolytic and hemicellulolytic enzymes, β-glucosidase, endoglucanase, exoglucanase, xylanase and glucoamylase by using sorghum husk as a substrate, thus leading to a reducing sugar production rate of 3.84 mg/h/L (Pankajkumar et al., 2018). *Sphingobacterium athyrii* sp. nov. isolated from a decaying fern (Athyrium wallichianum Ching) was also observed as gram-stain-negative, rod-shaped, nonmotile and aerobic with cellulose and xylan degradation abilities (Cheng et al., 2019). *Chryseobacterium culicis* Bp16 isolated from the Bandipur forest area, Karnataka, has also been reported as a potent cellulase producer (Kumar et al., 2023). The dominant bacterial in CK, such as *Paenibacillus*, has also been reported to produce glucanases, chitinases, cellulases, and proteases that are implicated in the destruction of eukaryotic cell walls (Grady et al., 2016).

The results confirmed that the *Bacillus* inoculant promoted *Lactobacillus* fermentation within 30 days of ensiling, which is why the lactic acid content was generally higher in the BA group. However, there were no significant differences in *Bacillus* proportions in the three groups at 30 days, indicating the slow growth of added *Bacillus* at the beginning of the ensiling process. Interestingly, at 60 days, the proportion of *Bacillus* in the BA group was significantly higher than that in the CK, which was probably related to the significant decrease of in NDF in the BA group at 60 days because *Bacillus* has the ability to degrade lignocellulose. In the BC group, the proportion of not only *Bacillus* but also the other bacteria were highest, and the synergy of these bacteria with cellulase possibly contributed to the higher NDF and ADF removal in the BC group.

#### 3.3.4 Bacterial enzyme shifts during ensiling

**Figure 6:**
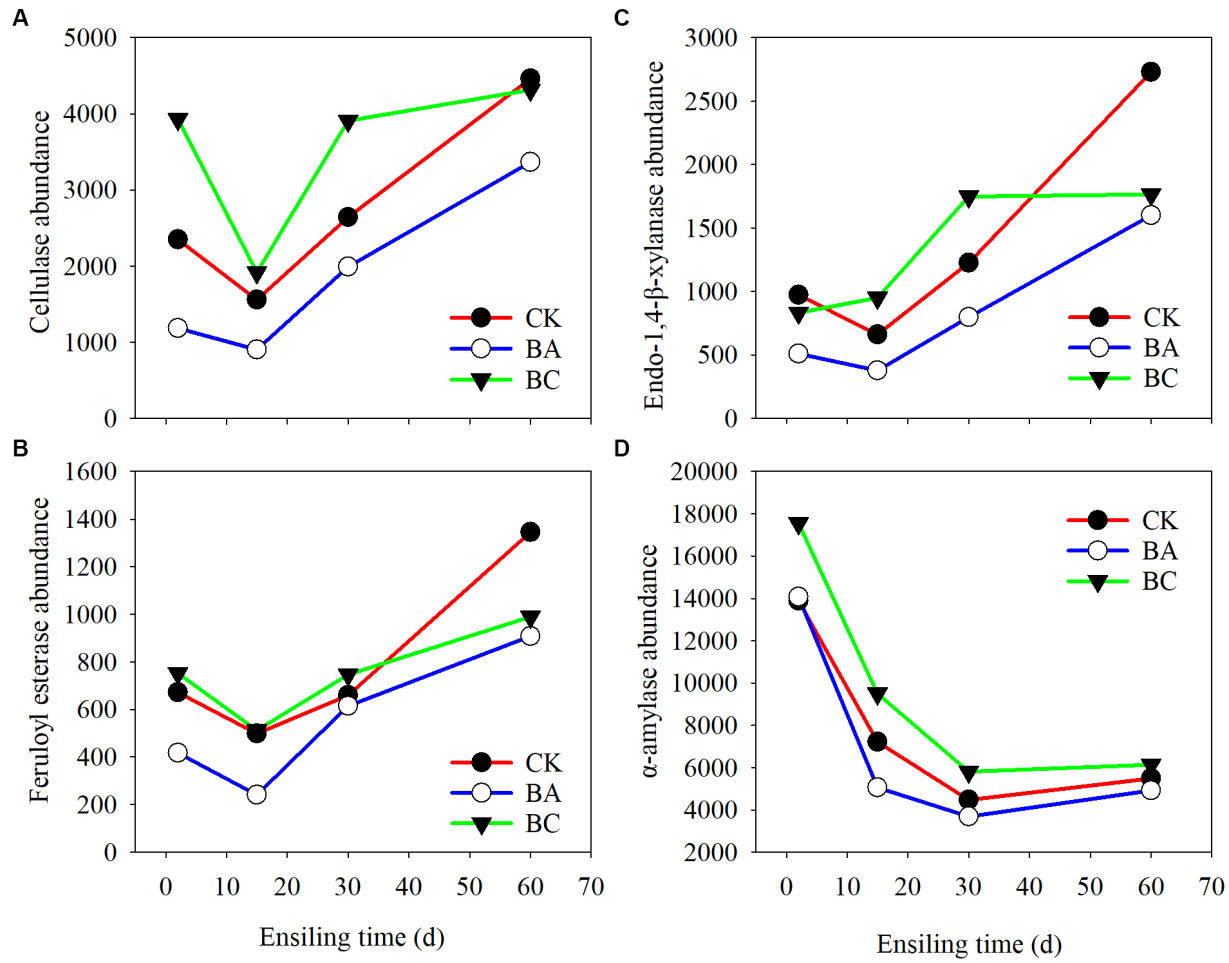
Bacterial alterations that contribute to abundance of enzyme shifts during the ensiling process. **(A)** cellulase; **(B)** endo-1,4-beta-xylanase; **(C)** feruloyl esterase; **(D)** α-amylase.

The enzymes of the bacterial communities of the three groups at different ensiling stages were predicted by PICRUSt2 software (Figure 6). The cellulase (EC:3.2.1.4), endo-1,4-beta-xylanase (EC:3.2.1.37), feruloyl esterase (EC:3.1.1.73), and α-amylase (EC:3.2.1.1) that are closely related to silage fermentation were chosen in the enzyme classification (EC) database. Overall, the abundances of cellulase, xylanase, and feruloyl esterase, which are regarded as the functional enzymes for lignocellulose degradation, decreased at 15 days of ensiling compared to those at 2 days (Figures 6A–C). However, their abundances increased afterward until 60 days. Basically, within 30 days, the abundances in the BC group were higher than those in the CK group, followed by the BA group. Interestingly, at 60 days, their abundances in the CK group exceeded the BC group. The abundances in BA groups consistently lower than the other two groups. The abundance of α-amylase, which is related to the starch degradation, were higher in BC groups at 2 days than in the CK and BA groups, and they all decreased in the following days of ensiling (Figure 6D).

The lignocellulose-degrading enzymes with high abundances in the three groups during the first 2 days of the ensiling probably acted on the decrease in NDF and ADF contents (Figure 2). The decrease in the abundances of these enzymes at 15 days was probably due to the significant increase in the abundance of *Lactobacillus*. As reported earlier, the dominance of LAB decreased alpha diversity (Xu et al., 2020). The enzyme abundances increased in the following days, which was due to the decrease in *Lactobacillus* and increase in *Bacillus* and other bacteria. However, in CK, no *Bacillus* was added as an inoculant; thus other bacteria, such as the observed *Paenibacillus*, could probably contributed to the increased enzyme activities. Correspondingly, there were more different bacteria in the BC group, which resulted in relatively higher enzyme activities for the degradation of NDF and ADF (Figure 5A). At 60 days, the abundances in CK increased rapidly to the highest, which could be due to the growth of bacteria, such as *Sphingobacterium* (Figure 5B).

The a-amylase during the first 2 days could probably act on starch degradation, as decreased starch contents were determined in the three groups. However, the abundances decreased in the following days, which can be explained by the increased abundances of bacteria for lignocellulose degradation. Of course, the chemical process for the degradation of NDF and ADF or starch cannot be ruled out because acidic conditions can also degrade carbohydrates (Larsen et al., 2017). As reported in an earlier study, the decrease in fiber content in *Bacillus subtilis* inoculated-silage might not be related to the abundance of cellulase (Bai et al., 2022).

Overall, it seems that there was a dynamic balance in the abundance of *Lactobacillus* and other bacteria, including the lignocellulolytic bacteria. The higher the abundance of *Lactobacillus* was, the lower abundance of the other bacteria. The *Bacillus* inoculant promoted the fast growth of *Lactobacillus* and suppressed the growth of other bacteria, leading to the rapid accumulation of lactic acid and little effect on NDF and ADF. Interestingly, the addition of cellulase helped the growth of lignocellulolytic bacteria, leading to the removal of NAD and ADF; and meanwhile, the degradation of NDF and ADF to ESC maintained the *Lactobacillus* fermentation until 60 days, leading to the significant accumulation of Lactic acid.

## 4 Conclusion

The *Bacillus* inoculant promoted fast lactic acid accumulation during the ensiling process of the WPCS. Although the addition of cellulase slowed lactic acid accumulation, it increased to highest level late. More importantly, cellulase significantly increased the degradation of NDF and ADF. The dynamic microbial community analysis suggested that the *Bacillus* inoculant seemed to have little effect on lignocellulose degradation. However, the addition of cellulase promoted microbial diversity, in which some potential lignocellulolytic microorganisms were observed, thus leading to more degradation of NDF and ADF. The results suggested that the addition of cellulase has potential to improve the nutritional quality of WPCS.

## Data availability statement

The data of 16S RNA sequences presented in this study are deposited in NCBI Bioproject repository, accession number PRJNA1035803.

## Author contributions

XL: Data curation, Formal analysis, Investigation, Writing – review & editing. AW: Data curation, Software, Validation, Writing – review & editing. LZ: Formal analysis, Resources, Writing – review & editing. WG: Methodology, Visualization, Writing – review & editing. XG: Conceptualization, Writing – review & editing. BZ: Supervision, Writing – review & editing. MY: Supervision, Writing – original draft, Writing – review & editing.
